# Factors Associated with Psychological Distress during COVID-19: A Cross-Sectional Study of Sub-Saharan African Migrant Workers across Australia and Canada

**DOI:** 10.3390/ijerph21091127

**Published:** 2024-08-27

**Authors:** Blessing J. Akombi-Inyang, Judith Byaruhanga, Sheila A. Boamah, John Allotey, Prince Atorkey

**Affiliations:** 1 School of Population Health, Faculty of Medicine and Health, University of New South Wales, Sydney, NSW 2052, Australia; 2 School of Medicine and Public Health, College of Health, Medicine and Wellbeing, University of Newcastle, Callaghan, NSW 2308, Australia; judith.byaruhanga@newcastle.edu.au (J.B.); or prince.atorkey@acap.edu.au (P.A.); 3 School of Nursing, Faculty of Health Sciences, McMaster University, Hamilton, ON L8S 4L8, Canada; boamahs@mcmaster.ca; 4 Institute of Metabolism and Systems Research (IMSR), University of Birmingham, Edgbaston, Birmingham B15 2TT, UK; j.allotey.1@bham.ac.uk; 5 Psychological Sciences, Australian College of Applied Professions (ACAP), Sydney, NSW 2000, Australia

**Keywords:** depression, anxiety, stress, mental health, pandemic, foreign workers, high-income countries, remittances, home country

## Abstract

**Objective**: Ensuring the sustainability of the migrant workforce requires a comprehensive understanding of the psychological challenges faced by this sub-population due to concerns about the wellbeing and financial situation of family members in their home countries. Therefore, this study investigates the factors associated with psychological distress among sub-Saharan Africa (SSA) migrant workers across Australia and Canada during the COVID-19 pandemic. **Method**: Data were collected from 378 first-generation migrant workers with SSA ancestry residing in Australia and Canada using the Depression Anxiety and Stress Scale 21 (DASS-21). Multivariate logistic regression analysis was used to determine socio-demographic factors associated with depression, anxiety, and stress among SSA migrants’ populations. **Results**: Across both countries, migrants with lower levels of education were more prone to reporting feelings of depression, anxiety, and stress during the pandemic. Female participants in Australia were more likely to report feeling of depression. Participants in Australia and Canada who were separated/divorced/widowed were less likely to report stress and depression, respectively. Participants in Australia who had lived in Australia between 11 and 20 years and those between 36 and 50 years old were more likely to report feelings of depression. Participants residing in Australia whose SSA ancestry was Southern Africa/Central Africa were more likely to report anxiety. Participants in Australia who worked as part-time permanent workers and those who worked as fixed-term workers/short-term/casual workers were less likely to report anxiety. Finally, participants in Canada who reported two or more people living with them had higher odds of reporting anxiety. **Conclusions**: The findings from this study highlight key factors associated with SSA migrant workers’ psychological distress during the pandemic. The results can inform policies and provide insight to the development of mental health intervention strategies for migrant workers to minimize similar distress during pandemics.

## 1. Background

The emergence of COVID-19 and the subsequent lockdowns had an impact on everyone to varying degrees [1]. However, the impact was significant among migrant populations including those of sub-Saharan African (SSA) ancestry in high-income countries (HICs). Most migrant workers from SSA in countries like Australia and Canada are the sole financial providers for their extended families in their home countries [2]. During the pandemic, many migrants struggled to secure employment due to the Government-imposed physical distancing measures [3]. Those who were working faced additional challenges of reduced work hours and wages, exacerbating financial pressures as they continued to send remittances to support their families in their home countries [4]. Migrants, often among the most marginalized sub-populations in society, heavily rely on daily wages for their livelihoods [5]. In times of distress, they require support and resources from the community to navigate their challenges [6].

Like migrants from other backgrounds, migrant workers of SSA ancestry are highly susceptible to experiencing social, psychological, and emotional trauma, especially during catastrophic events such as the COVID-19 pandemic [7,8]. Their concerns often revolve around the wellbeing and safety of their families, along with worries regarding access to necessities such as food, shelter, and healthcare. Additionally, they grapple with the fear of contracting or spreading infection, experiencing loss of wages/income, and increased anxiety [9]. The financial pressure coupled with the daily stressors of the pandemic may have severe long-term impacts on migrant workers’ mental and physical health and wellbeing.

The psychological distress of the COVID-19 pandemic may be exacerbated among populations already burdened by psychosocial factors, such as migrants, or people of temporary or undocumented immigration status, which already heighten their risk of psychiatric issues [10]. The stress of navigating the pandemic in a foreign country, combined with concerns for family back home, can lead to negative mental health outcomes [11]. Continuous stress without adequate coping mechanisms can have long-term psychological effects. Limited access to culturally sensitive mental health services can also hinder the ability of migrant workers to seek and receive appropriate care. Employment and financial difficulties are also frequently reported as contributing to negative psychological outcomes among immigrant populations [12]. Therefore, the exacerbation of social disparities and disproportionate job loss, when combined with the aforementioned levels of psychiatric distress, results in heightened psychological vulnerability for migrant populations [13].

While the travel restrictions and lockdowns helped to bring down the number of new infections, they worsened feelings of isolation, especially for those living away from family [14]. Limited social support networks in the host country can worsen loneliness and depression. Additionally, the uncertainty regarding visa statuses and fear of deportation can create additional stress. Frequent changes in immigration policies during the pandemic added to this uncertainty.

The International Organisation of Migration (IOM) released a set of guidelines [15] for employers and businesses to enhance migrant worker protection during the COVID-19 pandemic, with specific recommendations to address the unique vulnerabilities of migrant domestic workers. Recommendations included the adoption of health and safety measures in the home, the modification of work commutes to reduce the possibility of contracting or transmitting COVID-19, and the responsibilities of employers to ensure their domestic workers have up-to-date identification and migration documents. However, the guidelines did not address the mental health of migrant workers nor did the recommendations aim at mitigating the psychological distress caused by COVID-19 on migrant workers. To ensure the sustainability of the migrant workforce, it is essential to understand the psychological issues of this sub-population to inform targeted mental health programs. Therefore, this study will investigate the factors associated with psychological distress of the COVID-19 pandemic on SSA migrant workers across two countries: Australia and Canada. The findings from this study will inform policy as it pertains to the mental wellbeing of migrant workers in Australia and Canada, with application to migrant workers from SSA in other developed countries.

## 2. Method

A population-based cross-sectional survey was conducted to characterize the psychological distress caused by the COVID-19 pandemic on migrant workers with SSA ancestry. Data were collected from 378 first-generation migrant workers with SSA ancestry residing in Australia and Canada, resulting in an estimated sample size of 191, assuming a 95% confidence level, a 5% margin of error, and a 50% population proportion.

Australia and Canada were chosen for this study because SSA migration to these countries has increased due to economic opportunities, education, and family reunification. Australia has around 150,000 Sub-Saharan Africans, mostly in major cities, while Canada has about 250,000, primarily in urban centers. Both countries’ refugee programs also attract migrants from conflict-affected regions [16,17].

Migrant workers of SSA ancestry were recruited for the study via social media platforms (Facebook and WhatsApp) and emails advertising within community groups. Participants were eligible for the study if they were 18 years of age or older, employed (either on casual, part-time, or full-time basis) or self-employed, have lived in Australia or Canada for at least 1 year pre-COVID-19, and were proficient in the English language.

Using convenience sampling, a non-probability sampling technique where participants were selected based on their availability, ease of access, and proximity to the researcher, data were collected from April 2022 to August 2022 via an online questionnaire developed using Qualtrics under the following two sections: (i) socio-demographic characteristics; and (ii) psychological constructs (depression, anxiety, and stress). The online survey began with information about the study, informed consent, and details of the researchers. Participants provided consent by clicking ‘Yes’ after reading the details of the informed consent. Socio-demographic characteristics collected included the following: country of residence, level of education, sex, marital status, number of years lived abroad, age, country of birth, number of people living at home with them for the past 12 months, average earnings per annum, and employment status. For psychological constructs, a modified version of the Depression Anxiety Stress Scales (DASS)-21 tailored to the SSA migrant worker context was used. During this period, both countries were easing COVID-19 restrictions, but maintained some measures in high-risk areas and for international travel, likely affecting participant accessibility and behavior.

### 2.1. Depression Anxiety and Stress Scale 21 (DASS-21)

The DASS-21 is a self-report instrument designed to access mental health status [18]. The scale considers the adverse emotional states of depression, anxiety, and stress experienced by individuals and could be used in both clinical and non-clinical settings. DASS-21 has high internal consistency [19] and yields meaningful discriminations. It is used to measure current mental health state as well as changes in mental health state over time. The DASS is based on a dimensional rather than a categorical conception of psychological disorders, and scores emphasize the degree to which someone is experiencing symptoms rather than having diagnostic cut-off points. Participants were asked to respond on how closely the item applied to them in the past week. The scale uses the four-point Likert scoring system ranging from 0 (“did not apply to me at all”) to 3 (“applied to me most of the time”). The higher the score, the higher the level of negative emotions.

Given that this study was interested in examining psychological distress caused by financial distress and concern about the wellbeing of family overseas during COVID-19, some modifications were made to the original questions. For example, the first item on DASS 21 is “I found it hard to wind down” but this was modified to “I found it hard to wind down whenever I think of the wellbeing and financial situation of my family in my home country during the COVID-19 pandemic”.

### 2.2. Statistical Analysis

Data analysis was performed using STATA version 17 (Stata Corporation, College Station, TX, USA). Descriptive data are presented as frequencies and percentages for categorical data. Multivariate logistic regression was used to examine independent variables (i.e., socio-demographic characteristics) associated with dependent variables (i.e., depression, anxiety, and stress). Statistical significance was defined as two-tailed with *p*-value < 0.05.

## 3. Results

Table 1 shows the demographic characteristics of the participants. The majority (61.4%) of the participants resides in Australia, had college or tertiary as their highest education attainment (68.5%), and were aged 36–65 years (78.0%) (mean age = 40.9, SD = 7.7). More than half indicated their gender as female (55.6%), were currently married or cohabiting (66.9%), lived overseas for 1–10 years (53.4%), and 51.6% worked full-time. Approximately 42% indicated they were born in a West African country, 88.9% indicated they had two or more people living with them, 60% indicated their average earnings per annum to be between $37,001–90,000.

**Figure 1:**
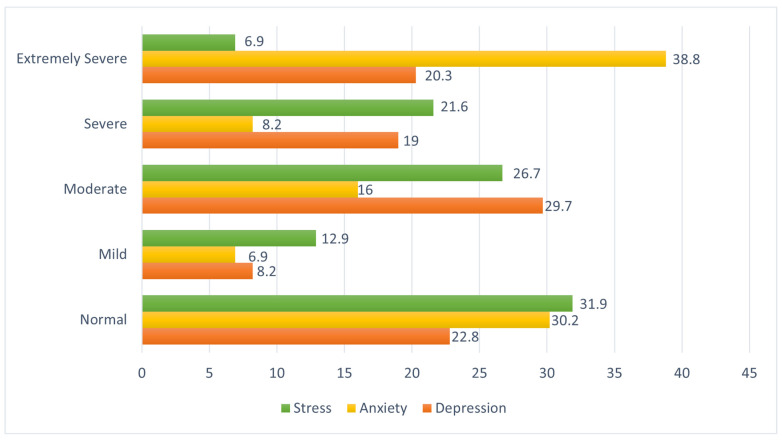
Prevalence of depression, anxiety, and stress among SSA migrant workers in Australia.

Figure 1 shows the prevalence of depression, anxiety and stress among participants in Australia. The prevalence of severe and extremely severe depression was 19% and 20.3%, respectively. The prevalence of severe and extremely severe anxiety was 8.2% and 38.8%, respectively. Finally, the prevalence of severe and extremely severe stress was 21.6% and 6.9%, respectively.

**Figure 2:**
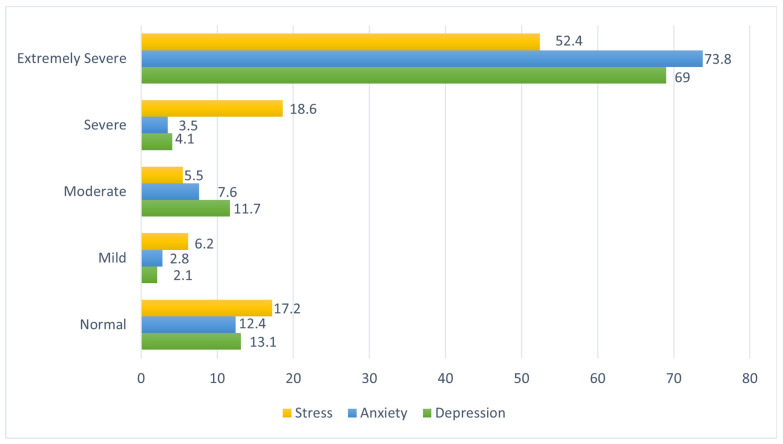
Prevalence of depression, anxiety, and stress among SSA migrant workers in Canada.

Figure 2 shows the prevalence of depression, anxiety, and stress among participants in Canada. The prevalence of severe and extremely severe depression was 4.1% and 69%, respectively. The prevalence of severe and extremely severe anxiety was 3.5% and 73.8%, respectively. Finally, the prevalence of severe and extremely severe stress was 18.6% and 52.4%, respectively.

**Figure 3:**
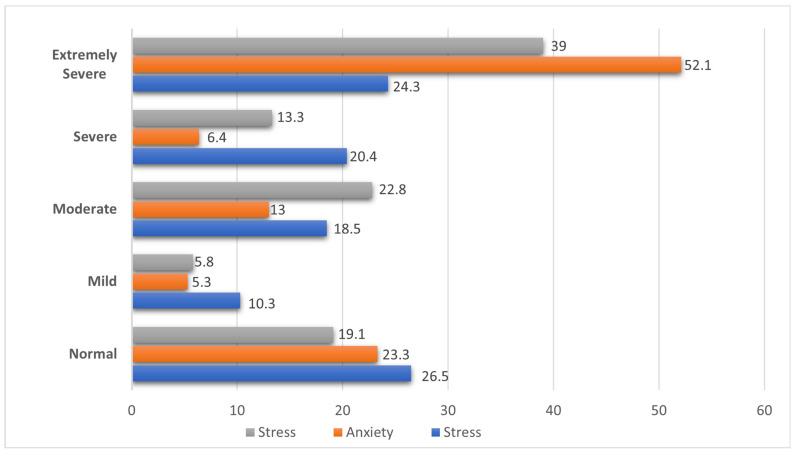
Prevalence of depression, anxiety, and stress among SSA migrant workers in Australia and Canada.

Figure 3 shows the prevalence of depression, anxiety, and stress among SSA migrant workers in Australia and Canada combined. About 22.8%, 13.3%, and 39% reported moderate, severe, and extremely severe symptoms of depression, respectively. In addition, 13%, 6.4%, and 52.1% reported they experienced moderate, severe, and extremely severe anxiety symptoms, respectively. Finally, 18.5%, 20.4%, and 24.3% reported moderate, severe, and extremely severe symptoms of stress, respectively.

### 3.1. Factors Associated with Depression, Anxiety, and Stress among Participants in Australia

Table 2 reports that people with college/tertiary education were less likely to report depression (AOR: 0.15; 95% CI 0.07, 0.35; *p* = 0.000), anxiety (AOR: 0.21; 95% CI 0.09, 0.49; *p* = 0.000), and stress (AOR: 0.21; 95% CI 0.09, 0.47; *p* = 0.000) compared to those with no formal schooling/less than primary school completed/primary or secondary school completed. Females were more likely to report symptoms of depression (AOR: 2.01; 95% CI 1.02, 3.98; *p* = 0.004). Participants who reported they were separated/divorced/widowed were more likely to report symptoms of stress (AOR: 4.16; 95% CI 1.22, 14.2; *p* = 0.023). Participants who had lived in Australia for 11–20 years were more likely to report symptoms of depression (AOR: 2.31; 95% CI 1.09, 4.89; *p* = 0.028). Also, participants who were 36 to 85 years were more likely to report symptoms of depression (AOR: 2.56; 95% CI 1.02, 6.43; *p* = 0.046). Participants from Southern African and Central African countries residing in Australia were more likely to report anxiety (AOR: 3.47; 95% CI 1.51, 7.98; *p* = 0.003). Fixed-term worker/short-term and casual workers were less likely to report symptoms of depression (AOR: 0.21; 95% CI 0.05, 0.81; *p* = 0.024). Part-time permanent workers (AOR: 0.16; 95% CI 0.04, 0.58; *p* = 0.006) and fixed-term worker/short-term and casual workers (AOR: 0.17; 95% CI 0.04, 0.68; *p* = 0.012) were less likely to report anxiety. 

### 3.2. Factors Associated with Depression, Anxiety, and Stress among Participants in Canada

Table 3 reports that participants with college/tertiary education were less likely to experience depression (AOR: 0.04; 95% CI 0.01, 0.26; *p* = 0.001), anxiety (AOR: 0.04; 95% CI 0.00, 0.34; *p* = 0.004), and stress (AOR: 0.47; 95% CI 0.23, 0.96; *p* = 0.037) compared to those with no formal schooling/less than primary school completed/primary or secondary school completed. Participants who indicated they were separated/divorced/widowed were less likely to report symptoms of depression (AOR: 0.29; 95% CI 0.00, 0.79; *p* = 0.036) compared to those who were never married. Finally, participants who indicated they had two or more people living with them were more likely to report anxiety (AOR: 12.68; 95% CI 1.39, 115.82; *p* = 0.024).

## 4. Discussion

This study reported the psychological impact of COVID-19 and associated factors on migrant workers with SSA ancestry across two countries of Australia and Canada. We found that, in general, migrants’ workers with SSA ancestry reported poor mental health during the pandemic due to concerns about the wellbeing and financial situation of their family in their home country. This is consistent with a previous review where 14 out of 26 studies [20] reported increased psychological distress among migrant workers during the pandemic, though the studies were mostly conducted in Latin and Asian countries. It is also important to note that our study population consisted of migrants, who have generally been found to be particularly vulnerable to mental health stressors even before the pandemic [21,22].

In this study, migrant workers with higher levels of education were found to be less likely to report experiencing depression, anxiety, and stress compared to those with lower educational attainment. However, as shown in previous studies, the impact of the COVID-19 pandemic on the mental health of migrant workers with college or tertiary education can vary greatly depending on various factors such as access to resources, social support networks, and pre-existing mental health conditions [23,24]. While higher education is typically associated with improved employment opportunities and socioeconomic status, it is crucial to acknowledge that the pandemic introduced distinct stressors and challenges for individuals with higher educational attainment. For instance, a previous study reported that some migrants experienced increased pressure to provide financial support to family members in their home countries, while their income in the host country remained unchanged [25]. This added financial responsibility could become a significant source of stress. Moreover, many educated individuals faced financial hardships during the pandemic, including job loss, reduced income, or difficulties in sending remittances back home, which is often a customary practice. Financial strains of this nature can exacerbate mental health issues such as depression, anxiety, and stress, especially for migrant workers with SSA ancestry who may already be grappling with supporting themselves and their extended families in their home countries.

In our research, migrant workers residing in Australia for 11–20 years and those aged between 36 and 65 years demonstrated a higher likelihood of reporting symptoms of depression during the pandemic. These findings were attributed to participants’ concern about the wellbeing and financial situation of their family in their home countries. Previous studies have indicated a correlation between older age and decreased resilience, as well as lower levels of mental health, particularly among displaced populations [22,26]. The COVID-19 pandemic has exposed vulnerabilities across various demographic groups, including older migrant worker populations. Understanding the interplay between older age and mental health, especially within migrant workers with SSA ancestry populations, requires careful examination. This subgroup may face disproportionate challenges such as job losses, economic instability, and difficulties accessing financial support or government assistance during the pandemic. Financial stressors, notably the pressure to send remittances back to their home countries, have been identified as significant contributors to mental health challenges, as evidenced in our study. Conversely, the Australian Institute of Health and Welfare’s (AIHW) 2020 report on mental health service utilization among younger Australians (aged 18–34 years) highlighted notably higher rates of mental health issues during the pandemic [27,28]. Remarkably, despite lacking the additional pressure of remittance obligations, this demographic still exhibited elevated levels of mental health concerns. However, existing data do not comprehensively illustrate the extent to which various cohorts of the Australian population, such as SSA migrant workers and refugees, have been impacted by the pandemic.

During the COVID-19 pandemic, female migrants in Australia exhibited a higher likelihood of reporting feelings of depression compared to their male counterparts. Migrant women may have faced increased pressure to send remittances during the pandemic because females are generally viewed as been pliable and emotional givers. This added financial responsibility could contribute to stress and anxiety for migrant women, particularly if they were experiencing financial difficulties themselves due to job loss, reduced income, or other economic challenges brought about by the pandemic. This trend aligns with findings from prior studies focusing on refugee and migrant women from low-income countries in the SSA region. These studies highlighted that these women were already vulnerable to negative mental health outcomes before the pandemic [29]. Moreover, additional research has suggested that the pandemic exacerbated gender disparities in health risks, with women being disproportionately affected by certain adverse health outcomes, including mental health issues. This underscores the importance of recognizing and addressing the specific challenges faced by female migrants, particularly during times of crisis such as the COVID-19 pandemic [29,30,31].

In this study, participants who indicated they had two or more people living with them were more likely to report anxiety in Canada during the pandemic. This could be a concern related to financial worries for basic needs such as housing and food, especially in the face of joblessness and the need to provide for their nuclear family as well as send remittances to their extended family in their home country during the strict lockdowns [32]. Similarly, participants who reported they were separated, divorced, or widowed, were more likely to report symptoms of stress in Australia during the pandemic. This may be due to heightened feelings of loneliness and social isolation during the pandemic, especially if they live alone or have limited social networks. Social distancing measures and restrictions on gatherings can exacerbate these feelings, as they may not have the same level of in-person social interaction or support as those in coupled relationships [33]. In addition, separated, divorced, or widowed individuals may face greater financial strain during the pandemic, particularly if they are the sole breadwinner for their family in their home country or if they are reliant on alimony or survivor benefits. Single income or reduced work hours can lead to financial strain, which in turn can contribute to anxiety, depression, and other mental health issues [34]. The COVID-19 pandemic highlighted the importance of targeted support and interventions to address the unique needs of these individuals including providing access to mental health services, financial assistance, and social support networks.

## 5. Strengths and Limitations

This study focused on SSA migrant workers in Australia and Canada, addressing a unique and under-researched population. Including participants from both countries allows for comparative analysis across different sociocultural and healthcare contexts. Data collected during a significant period of the COVID-19 pandemic captured relevant impacts within this population. By gathering both socio-demographic and psychological data, the study provides a holistic view of participants’ experiences. With 378 participants, the study offers a robust dataset for analyzing psychological impacts while the use of multivariate logistic regression ensured thorough examination of associations between socio-demographic factors and psychological outcomes. Utilizing the validated DASS-21 tool enhances the reliability of measuring depression, anxiety, and stress, with modifications to suit SSA migrant workers’ context.

Despite these strengths, this study had some limitations. First, the sample size of 378, while adequate for the study design, may limit the generalizability of the findings to all SSA migrant workers in Australia and Canada. The sample may not fully represent the diverse experiences and backgrounds within this population. Second, recruitment via social media within community groups may have introduced selection bias. Individuals active in such groups may have different experiences and mental health outcomes compared to those not engaged in these online communities. Third, the reliance on self-reported data for psychological constructs can introduce response bias. Participants may have underreported or overreported symptoms due to social desirability or recall biases. Fourth, conducting the survey online and among only participants proficient in English may have excluded individuals with limited internet access or lower digital literacy, potentially skewing the sample towards more technologically adept individuals as well as excluding non-English-speaking SSA migrant workers, thus limiting the diversity of the sample and the applicability of the findings to all SSA migrant workers. Fifth, while the DASS-21 was modified to consider the SSA migrant worker context, the modifications may have affected the validity and reliability of the scale compared to its original version. In addition, the study relied solely on quantitative measures, which may not capture the nuanced, subjective experiences and coping mechanisms of SSA migrant workers during the COVID-19 pandemic. Furthermore, data were collected between April 2022 and August 2022, a period that may not capture the full range of psychological impacts of COVID-19, especially as the pandemic’s situation and associated stressors evolved. It is also worth noting that although ethical clearance was obtained, the study may not have fully addressed cultural sensitivities and specific contextual factors affecting the SSA migrant worker population’s mental health. Finally, the cross-sectional nature of the survey limits the ability to infer causality. The data provide a snapshot of psychological impacts during the specified period but do not track changes over time.

## 6. Policy Implications

This research serves as a crucial initial exploration into the determinants of mental health struggles encountered by migrants from SSA during the COVID-19 pandemic. Its findings will help policymakers better understand the urgent need to allocate mental health support and resources to these communities. This includes ensuring access to counselling services, financial aid, and avenues for social interaction and support. Ultimately, the insights gleaned from this research will inform the development of targeted mental health intervention strategies, aimed at mitigating the adverse effects of the pandemic on migrant workers populations.

## 7. Conclusions

This study examined the psychological impact of COVID-19 on migrant workers with SSA ancestry in Australia and Canada. These workers reported poor mental health due to stressors from their home countries. Higher education correlated with lower depression, anxiety and stress, but financial pressures remained significant. Older migrants and those in Australia for 11–20 years reported higher depression rates, exacerbated by financial stress. Female migrants experienced increased depression due to financial pressures. In Canada, living with multiple people heightened anxiety, while separated or widowed individuals in Australia reported more stress. Targeted support and interventions are needed to address these mental health challenges.

## Figures and Tables

**Table 1:** Demographic characteristics.

Variables	N	%
**Country of Residence**		
Canada	146	38.5
Australia	232	61.4
**Highest level of Education**		
No formal schooling/Less than primary school completed/Primary school completed/Secondary school completed	119	31.5
College/Tertiary	259	68.5
**Gender**		
Male	168	44.4
Female	210	55.6
**Marital Status**		
Never Married	75	19.8
Currently Married/Cohabiting	253	66.9
Separated/Divorced/Widowed	50	13.2
**Number of Years Lived Abroad**		
1–10 years	202	53.4
11–20 years	127	33.6
21–38 years	49	13.0
**Age**		
18–35 years	80	21.2
36–65 years	295	78.0
**Sub-Saharan Regions**		
East Africa	130	34.4
West Africa	159	42.1
Southern Africa/Central Africa	53	23.5
**Number of people living with**		
Alone	42	11.1
Two or more	336	88.9
**Average Earnings (per annum)**		
$0–37,000	32	8.5
$$37,001–90,000	226	59.8
$90,001 and above	120	31.8
**Employment Status**		
Self-employed	44	11.6
Full-time worker	195	51.6
Part-time permanent worker	85	22.5
Fixed-term worker/Short-term and Casual worker	54	14.3

**Table 2:** Factors associated with depression, anxiety, and stress among participants in Australia.

Variables	Depression			Anxiety			Stress		
	AOR	*p*-Value	95% CI	AOR	*p*-Value	95% CI	AOR	*p*-Value	95% CI
**Highest level of Education**									
No formal schooling/Less than primary school completed/Primary school completed/Secondary school completed	1			1			1		
College/Tertiary	0.15	**0.000**	0.07, 0.35	0.21	**0.000**	0.09, 0.49	0.21	**0.000**	0.09, 0.47
**Gender**									
Male	1			1			1		
Female	2.01	**0.004**	1.02, 3.98	1.42	0.297	0.74, 2.74	1.30	0.467	0.64, 2.67
**Marital Status**									
Never Married	1			1			1		
Currently Married/Cohabiting	0.83	0.712	0.32, 2.19	1.00	0.997	0.39, 2.58	0.83	0.721	0.30, 2.28
Separated/Divorced/Widowed	1.92	0.287	0.58, 6.35	1.60	0.448	0.47, 5.41	4.16	**0.023**	1.22, 14.2
**Number of Years Lived Abroad**									
1–10 years	1			1			1		
11–20 years	2.31	**0.028**	1.09, 4.89	1.77	0.139	0.83, 3.75	2.10	0.067	0.95, 4.63
21–38 years	1.25	0.701	0.40, 3.93	0.99	0.984	0.31, 3.12	1.11	0.869	0.33, 3.70
**Age**									
18–35 years	1			1			1		
36–65 years	2.56	**0.046**	1.02, 6.43	1.05	0.916	0.44, 2.47	1.90	0.201	0.71, 5.04
**Sub-Saharan Regions**									
East Africa	1			1			1		
West Africa	1.15	0.723	0.54, 2.43	1.01	0.969	0.49, 2.11	1.18	0.689	0.53, 2.65
Southern Africa/Central Africa	1.28	0.571	0.55, 2.96	3.47	**0.003**	1.51, 7.98	1.28	0.586	0.53, 3.08
**Number of people living with**									
One	1			1			1		
Two and more	1.15	0.819	0.35, 3.75	1.52	0.470	0.49, 4.68	0.99	0.992	0.29, 3.39
**Average Earnings**									
$0–37,000	1			1			1		
$37,001–90,000	0.56	0.345	0.16, 1.88	1.26	0.695	0.40, 3.99	0.80	0.717	0.23, 2.71
$90,001 and above	0.44	0.252	0.11, 1.80	0.53	0.362	0.14, 2.07	0.38	0.212	0.09, 1.72
**Employment Status**									
Self-employed	1			1			1		
Full-time worker	0.52	0.236	0.18, 1.53	0.31	0.052	0.09, 1.01	1.15	0.798	0.40, 3.30
Part-time permanent worker	0.47	0.221	0.14, 1.57	0.16	**0.006**	0.04, 0.58	0.87	0.822	0.27, 2.83
Fixed-term worker/Short-term and Casual worker	0.21	**0.024**	0.05, 0.81	0.17	**0.012**	0.04, 0.68	0.76	0.682	0.20, 2.83

**Table 3:** Factors associated with depression, anxiety, and stress among participants in Canada.

Variables	Depression			Anxiety			Stress		
	AOR	*p*-Value	95% CI	AOR	*p*-Value	95% CI	AOR	*p*-Value	95% CI
**Highest level of Education**									
No formal schooling/Less than primary school completed/Primary school completed/Secondary school completed	1			1			1		
College/Tertiary	0.04	**0.001**	0.01, 0.26	0.04	**0.004**	0.00, 0.34	0.27	**0.000**	0.00, 0.19
**Gender**									
Male	1			1			1		
Female	0.64	0.392	0.23, 1.79	0.49	0.197	0.16, 1.45	0.67	0.449	0.24, 1.89
**Marital Status**									
Never Married	1			1			1		
Currently Married/Cohabiting	0.19	0.118	0.02, 1.52	0.12	0.066	0.12, 1.15	0.17	0.109	0.02, 1.47
Separated/Divorced/Widowed	0.29	**0.036**	0.00, 0.79	0.30	0.468	0.12, 7.57	0.05	0.067	0.00, 1.23
**Number of Years Lived Abroad**									
1–10 years	1			1			1		
11–20 years	1.12	0.861	0.32, 3.89	1.02	0.971	0.28, 3.70	1.49	0.524	0.44, 5.10
21–38 years	4.34	0.272	0.32, 59.48	2.65	0.455	0.20, 34.5	4.03	0.278	0.32, 49.10
**Age**									
18–35 years	1			1			1		
36–65 years	2.13	0.268	0.56, 8.12	1.79	0.408	0.45, 7.15	2.00	0.296	0.54, 7.42
**Sub-Saharan Regions**									
East Africa	1			1			1		
West Africa	0.36	0.131	0.09, 1.36	0.40	0.222	0.09, 1.75	0.26	0.051	0.07, 1.00
Southern Africa/Central Africa	3.60	0.202	0.50, 25.81	2.68	0.350	0.34, 21.2	2.11	0.410	0.36, 12.39
**Number of people living with**									
One	1			1			1		
Two and more	8.63	0.052	0.98, 75.62	12.68	**0.024**	1.39, 115.82	7.89	0.075	0.81, 76.42
**Average Earnings**									
$0–37,000	1			1			1		
$37,001–90,000	0.74	0.827	0.05, 10.81	1.56	0.749	0.10, 23.32	0.39	0.488	0.03, 5.50
$90,001 and above	0.41	0.525	0.03, 6.24	0.62	0.728	0.04, 9.42	0.211	0.258	0.01, 3.13
**Employment Status**									
Self-employed	1			1			1		
Full-time worker	0.31	0.448	0.02, 6.39	0.76	0.868	0.03, 19.33	0.33	0.486	0.01, 7.62
Part-time permanent worker	0.17	0.269	0.01, 3.85	0.54	0.715	0.02, 14.60	0.19	0.316	0.01, 4.86
Fixed-term worker/Short-term and Casual worker	0.65	0.818	0.02, 26.02	4.07	0.527	0.05, 315.98	0.08	0.185	0.00, 3.31

## Data Availability

The data presented in this study are available on request from the corresponding author. The data are not publicly available due to privacy or ethical restrictions.
